# Page Kidney in a Child with Severe Pelviureteric Junction Obstruction

**DOI:** 10.1155/2020/8883546

**Published:** 2020-08-20

**Authors:** Senthil G. Kamaraj, Ashita Kochunny, Gopinathan Kathirvelu, Praachi Singh, Naeem Samnakay

**Affiliations:** ^1^Consultant Paediatric Urologist, Kanchi Kamakoti Child's Trust Hospital, 12-A, Nageswara Road, Nungambakkam, Chennai 600034, India; ^2^Registrar in Paediatric Surgery, Kanchi Kamakoti Child's Trust Hospital, India; ^3^Consultant Radiologist, Kanchi Kamakoti Child's Trust Hospital, India; ^4^Registrar in Paediatrics, Kanchi Kamakoti Child's Trust Hospital, India; ^5^Clinical Associate Professor, University of Western Australia, Paediatric Urologist, Perth Children's Hospital, 12 Hospital Ave, Nedlands, WA 6009, Australia

## Abstract

There are various causes of Reno Vascular Hypertension in children reported in the literature. Amongst these, Page kidney gets a rare mention. This phenomenon is a result of the accumulation of blood or urine in the perinephric or subcapsular space, resulting in compression of renal parenchyma, microvascular ischemia, alteration in the renin-angiotensin apparatus, and high renin hypertension. It has been well documented and studied in adults. Only a few cases are reported in the paediatric population. We report a rare presentation of Page kidney in a 5 year 8 months old girl. She initially presented with Dietl's crisis secondary to left Pelviureteric Junction obstruction (PUJO) causing massive hydronephrosis. She developed Page kidney phenomenon after spontaneous rupture of the pelvicalyceal system formed a tight compressive urinoma. She was managed successfully with internal JJ stenting and ultrasound-guided aspiration of the urinoma followed by elective delayed Pyeloplasty. To our knowledge, this is the first documented case of Page kidney in a child with severe PUJO.

## 1. Introduction

Page kidney refers to the occurrence of hypertension secondary to renal compression and is usually associated with a subcapsular or perinephric hematoma [1]. The first documented clinical case of Page kidney was reported in 1955 by Engel and Page [2]. Thereafter, many cases were reported, mainly in adults following trauma. We report a case of Page kidney in a child with PUJO, developed after spontaneous rupture of the pelvicalyceal system formed a massive subcapsular urinoma.

## 2. Case Report

**Figure 1:**
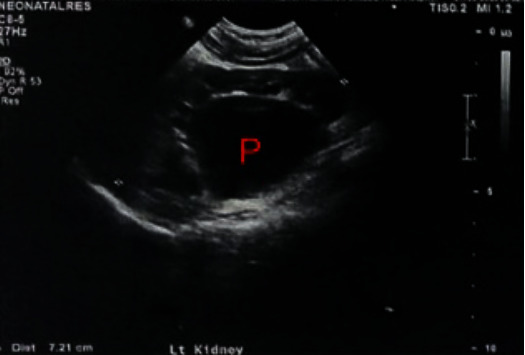
Ultrasound showing severe left PUJO at presentation.

A 5 year 8 month old presented to the emergency department with abdominal pain and non bilious vomits for 4 days. On clinical examination, there was a palpable loin mass. Ultrasound (USS) showed marked left (Figure 1) hydronephrosis with thinning of the renal cortex and severely dilated renal pelvis with an anteroposterior diameter of 6.3 cm. She was admitted to the ward for further assessment and management. EC scan, done with free draining bladder catheter, showed a differential function of 22% in the left kidney: with an obstructive drainage pattern. The Right kidney had 78% split function and a normal drainage pattern. An early pyeloplasty during the same admission was planned.

Overnight, the girl developed seizures and elevated blood pressure of 120/78 (99^th^ centile for age). In this acute setting, she was shifted to the Intensive care unit where she was managed with antihypertensive (Amlodipine and Prazosin) and anticonvulsant medications (Lorazepam and Levetiracetam). MRI Brain showed changes secondary to Malignant Hypertension and ruled out bleeding or infarction. With treatment, her seizures were controlled and stabilisation of blood pressure was achieved.

**Figure 2:**
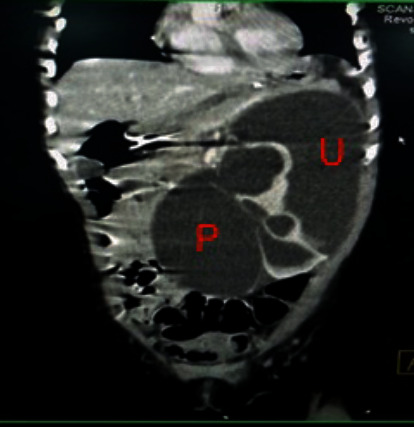
CT showing severe PUJO, ruptured with compressive urinoma. P: pelvis, U: compressive urinoma.

A repeat USS showed a large perinephric/subcapsular collection of size 18.3x10.0 cm with internal septae encompassing the left kidney. CT scan demonstrated a left PUJO associated with extravasation of contrast from the left pelvicalyceal system and dilated pelvis, forming a 13x10x6.5 cm large subcapsular collection with internal hemorrhage. This collection resulted in displacement of the left kidney and compression of left renal parenchyma—The clinical and imaging features were suggestive of Page kidney (Figure 2).

**Figure 3:**
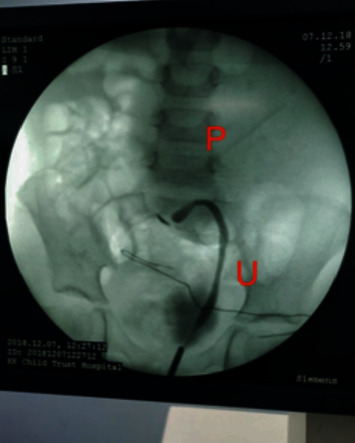
Retrograde study showing left PUJO with diluted contrast in the massive pelvis (P) and contrast in the left ureter.

**Figure 4:**
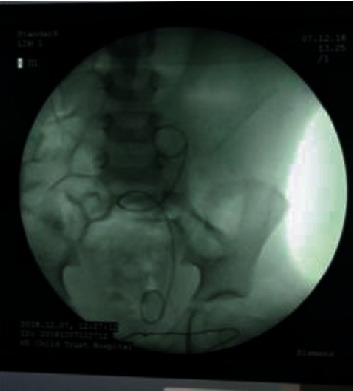
Xray KUB showing JJ stent across the left PUJ.

**Figure 5:**
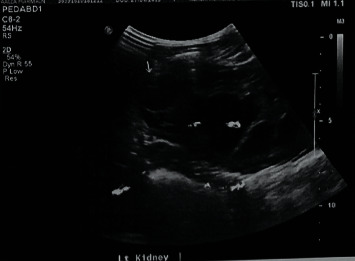
Day 5 post-JJ stenting ultrasound showing resolution of urinoma and decompression of the left kidney.

Under general anaesthetic, she underwent USS guided aspiration of the tense subcapsular collection (150 ml), followed by a Retrograde Pyelogram (RGP) and cystoscopic placement of a 3.5 F 16 cm JJ stent across the PUJO (Figures 3 and 4). She stabilised postprocedure with normalisation of blood pressure and control of seizures. Repeat USS 5 days later showed resolving perinephric collection and reduction in hydronephrosis (Figure 5). She was discharged home on antibiotic prophylaxis. The antihypertensive and antiepileptic medications were successfully tapered and ceased in the hospital prior to discharge.

Six weeks later, repeat USS showed complete resolution of the subcapsular collection and further reduction in APD of the left renal collecting system to 1.1 cm. In order to reassess the split renal function on the left, a DMSA scan was performed; Split renal function of left kidney now, with relief of acute obstruction, was 42% as compared to 22% before the stenting and drainage. She underwent a Left Anderson Hynes dismembered Pyeloplasty. The postoperative period was uneventful. JJ stent was removed 6 weeks postsurgery.

After a year of follow-up, she has been symptom-free, off medications and has minimal residual hydronephrosis on USS.

## 3. Discussion

The term Page kidney originated in 1939 when Dr. Irvin Page performed an experiment in which he wrapped the kidneys of dogs in cellophane [3]. He observed changes of perinephritis and the development of hypertension in these dogs. Hypertension develops due to external compression of renal parenchyma leading to microvascular ischemia and activation of Renin-Angiotension-Aldosterone System [1]. A similar response occurs when subcapsular or perinephric hematoma or encasing fibrous capsule causes induction of perinephritis and parenchymal compression [4].

Our case already had a pelvicalyceal (PCS) system under severe pressure, and after rupture of the PCS, there was in addition a tense external urinoma compressing the parenchyma and renal hilum further.

There have been many reports of Page kidney occurring following trauma in adults [5]. Other causes of Page kidney are tumour, arteriovenous malformation, cyst rupture, glomerulonephritis, vasculitis and native kidney biopsy, and spontaneous intrarenal haemorrhage [5, 6]. There are few reports of children with Page kidney [7]. Contrast-enhanced CT is the preferred modality to diagnose subcapsular or perinephric renal hematoma [3]. Surgical treatment is the mainstay of management including minimally invasive procedures like image-guided percutaneous aspiration of hematoma [8]. Nephrectomy may be necessary in refractory cases. Antihypertensives of choice in Page kidney induced hypertension are the Angiotensin Converting Enzyme (ACE) Inhibitors [4, 6]. Generally, complete resolution of hypertension [3] follows drainage of the perirenal fluid.

Our case developed malignant hypertension after spontaneous rupture of the pelvicalyceal system, with compression of the left renal parenchyma from the resultant tense subcapsular hematoma. The hypertension settled after ultrasound-guided drainage of the subcapsular urinoma and internal drainage of the obstructed left kidney by JJ stent across the PUJO. Repeat ultrasound scan showed complete resolution of the subcapsular urinoma after 6 weeks, after which the child underwent an uneventful pyeloplasty.

Spontaneous rupture of the pelvicalyceal system is rare in PUJO. In our case, symptoms of loin pain followed by rapid development of malignant hypertension lead to the early diagnosis of Page phenomenon, resulting in prompt successful management,avoiding the morbidity of prolonged malignant hypertension. A high index of suspicion is required to diagnose Page kidney in children with suspected PUJO who develop acute or recent onset hypertension.

## 4. Conclusion

Page kidney is rare in children and generally posttraumatic in origin. Our case demonstrates Page kidney can occur in the setting of severe PUJO. It should be suspected early in children with suspected PUJO with acute onset hypertension.Prompt management by drainage of perinephric or subcapsular urino-hematoma, as well as internal drainage of the PUJO with JJ stenting, will help in the resolution of Page kidney hypertension and prevent further deterioration of renal function.
